# Machine Learning: An Overview and Applications in Pharmacogenetics

**DOI:** 10.3390/genes12101511

**Published:** 2021-09-26

**Authors:** Giovanna Cilluffo, Salvatore Fasola, Giuliana Ferrante, Velia Malizia, Laura Montalbano, Stefania La Grutta

**Affiliations:** 1 Institute for Biomedical Research and Innovation, National Research Council, 90146 Palermo, Italy; salvatore.fasola@irib.cnr.it (S.F.); velia.malizia@irib.cnr.it (V.M.); laura.montalbano@irib.cnr.it (L.M.); stefania.lagrutta@irib.cnr.it (S.L.G.); 2 Department of Surgical Sciences, Dentistry, Gynecology and Pediatrics, Pediatric Division, University of Verona, 37134 Verona, Italy; giuliana.ferrante@univr.it

**Keywords:** pharmacogenetics, supervised machine learning, unsupervised machine learning

## Abstract

This narrative review aims to provide an overview of the main Machine Learning (ML) techniques and their applications in pharmacogenetics (such as antidepressant, anti-cancer and warfarin drugs) over the past 10 years. ML deals with the study, the design and the development of algorithms that give computers capability to learn without being explicitly programmed. ML is a sub-field of artificial intelligence, and to date, it has demonstrated satisfactory performance on a wide range of tasks in biomedicine. According to the final goal, ML can be defined as Supervised (SML) or as Unsupervised (UML). SML techniques are applied when prediction is the focus of the research. On the other hand, UML techniques are used when the outcome is not known, and the goal of the research is unveiling the underlying structure of the data. The increasing use of sophisticated ML algorithms will likely be instrumental in improving knowledge in pharmacogenetics.

## 1. Introduction

Pharmacogenetics aims to assess the interindividual variations in DNA sequence related to drug response [1]. Gene variations indicate that a drug can be safe for one person but harmful for another. The overall prevalence of adverse drug reaction-related hospitalization varies from 0.2% [2] to 54.5% [3]. Pharmacogenetics may prevent drug adverse events by identifying patients at risk in order to implement personalized medicine, i.e., a medicine tailored focused on genomic context of each patient. 

The need to obtain increasingly accurate and reliable results, especially in pharmacogenetics, is leading to a greater use of sophisticated data analysis techniques based on experience called Machine Learning (ML). ML can be defined as the study of computer algorithms that improve automatically through experience. According to Tom M. Mitchell “A computer program is said to learn from experience E with respect to some class of tasks T and performance measure P if its performance at tasks in T, as measured by P, improves with experience E.” [4]. According to the final goal, ML can be defined as Supervised (SML) or as Unsupervised (UML). SML techniques are applied when prediction is the focus of the research. On the other hand, UML techniques are used when the outcome is not known, and the goal of the research is unveiling the underlying structure of the data. 

**Figure 1:**
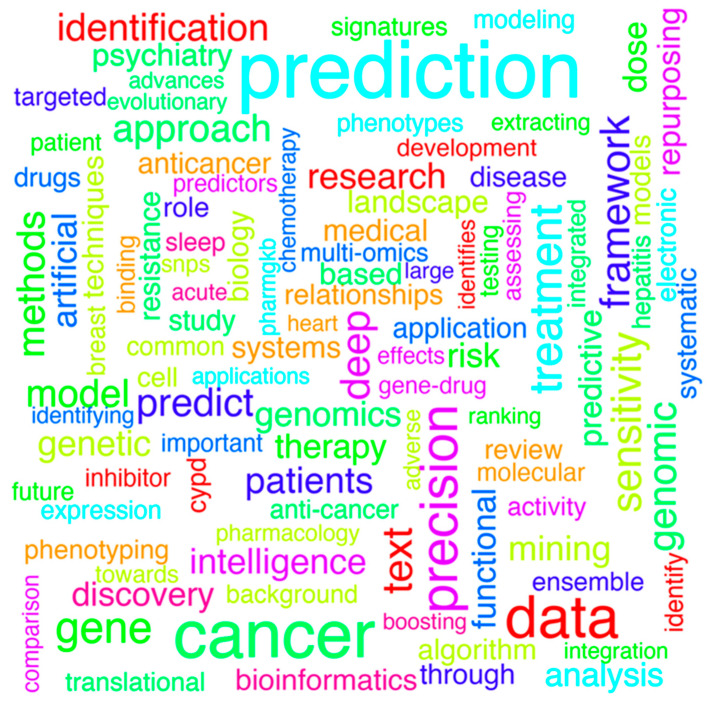
Word-cloud analysis using the titles of articles obtained based on the following search strategy (PubMed): machine learning AND pharmacogenetics. The pre-processing procedures applied were: (1) removing non-English words or common words that do not provide information; (2) changing words to lower case and (3) removing punctuation and white spaces. The size of the word is proportional to the observed frequency.

This narrative review aims to provide an overview of the main SML and UML techniques and their applications in pharmacogenetics over the past 10 years. The following search strategy, with a filter on the last 10 years, was run on PubMed “machine learning AND pharmacogenetics” (Figure 1).

The paper is organized as follows: Section 2 illustrates the SML approach and its application on pharmacogenetics; Section 3 reports the principal UML approach and its application on pharmacogenetics; Section 4 is devoted to discussion.

## 2. Supervised Machine Learning Approaches

**Figure 2:**
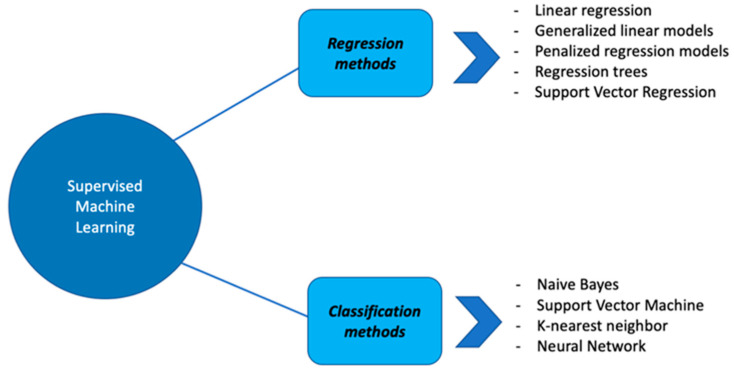
Summary representation of different SML algorithms: examples of regression and classification methods.

Several SML techniques have been implemented. They can be classified into two categories: regression methods and classification methods (Figure 2).

### 2.1. Regression Methods

The simplest regression method is linear regression. A linear model assumes a linear relationship between the input variables (*X*) and an output variable (*Y*) [5]. Standard formulation of linear regression models with standard estimation techniques is subject to four assumptions: (i) linearity of the relationship between *X* and expected value of *Y*; (ii) homoscedasticity, i.e., the residual variance is the same for any value of *X*; (iii) independence of the observations and (iv) normality: the conditional distribution of *Y*|*X* is normal. To overcome the linear regression model assumptions, the generalized linear models (GLM) have been developed. The GLM generalize linear regression by allowing the linear model to be related to the response variable via a link function [6,7]:

$$E\left(Y|X\right)=\mu_{i}=g^{-1}\left(x_{i}^{T}\beta \right)$$

where $\mu_{i}$ is the response function, and g is the link function.

In order to address more complex problems, sophisticated penalized regression models have been developed allowing to overcome problems such as multicollinearity and high dimensionality. In particular, Ridge regression [8] is employed when problems with multicollinearity occur, and it consists of adding a penalization term to the loss function as follows:

$$\underset{\beta }{\arg\min}\|y-X\beta \|+\lambda {\left\|\beta \right\|}_{2}^{2}$$

where λ is the amount of penalization (tuning parameter), and ${\left\|\beta \right\|}_{2}^{2}$ is the norm 2 of the βs, i.e., ${\left\|\beta \right\|}_{2}^{2}=\sum \beta_{i}^{2}$. More recently, Tibshirani et al. introduced LASSO regression, an elegant and relatively widespread solution to carry out variable selection and parameter estimation simultaneously, also in high dimensional settings [9]. In LASSO regression, the objective function to be minimized is the following:

$$\underset{\beta }{\arg\min}\|y-X\beta \|+\lambda {\left\|\beta \right\|}_{1}$$

where λ is the amount of penalization (tuning parameter), and ${\left\|\beta \right\|}_{1}$ is the norm 1 of the βs, i.e., ${\left\|\beta \right\|}_{1}=\sum \beta_{i}$. Some issues concerning the computation of standard errors and inference have been recently discussed [10]. A combination of LASSO and Ridge regression penalties leads to the Elastic Net (EN) regression:

$$\underset{\beta }{\arg\min}\|y-X\beta \|+\lambda_{1}{\left\|\beta \right\|}_{1}+\lambda_{2}{\left\|\beta \right\|}_{2}^{2}$$

where $\lambda_{1}{\left\|\beta \right\|}_{1}$ is the L1 penalty (LASSO), and $\lambda_{2}{\left\|\beta \right\|}_{2}^{2}$ is the L2 penalty (Ridge). Regularization parameters reduce overfitting, decreasing the variance of the estimated regression parameters; the larger the λ, the more shrunken the estimate; however, more bias will be added to the estimates. Cross-Validation can be used to select the best value of λ to use in order to ensure the best model is selected. Another family of regression methods is represented by regression trees. A regression tree is built by splitting the whole data sample, constituting the root node of the tree, into subsets (which constitute the successor children), based on different cut-offs on the input variables [11]. The splitting rules are based on measures of prediction performances; in general, they are chosen to minimize the residual sum of squares:

$$RSS=\overset{n}{\underset{i=1}{\sum }}{\left(y_{i}-\hat{y_{i}}\right)}^{2}$$

The pseudo algorithm works as follows:

1. Start with a single node containing all the observations. Calculate $\hat{y_{i}}$ and *RSS*;
2. If all the observations in the node have the same value for all the input variables, stop. Otherwise, search over all binary splits of all variables for the one which will reduce *RSS* as much as possible;
3. Restart from step 1 for each new node.

Random forests (RF) are an ensemble learning method based on a multitude of decision trees; to make a prediction for new input data, the predictions obtained from each individual tree are averaged [12].

RuleFit is another ensemble method that combines regression tree methods and LASSO regression [13]. The structural model takes the form:

$$F\left(x\right)=a_{0}+\overset{M}{\underset{m=1}{\sum }}a_{m}f_{m}\left(x\right)$$

where *M* is the size of the ensemble and each ensemble member (“base learner”), and $f_{m}\left(x\right)\;$ is a different function (usually the indicator function) of the input variables x. Given a set of base learners $f_{m}\left(x\right)$, the parameters of the linear combination are obtained by

$$\;{\left\{{\hat{a}}_{m}\right\}}_{0}^{M}=\arg\underset{{\left\{a_{m}\right\}}_{0}^{M}}{\min}\overset{N}{\underset{i=1}{\sum }}L\left(y_{i},\;F\left(x\right)\right)+\lambda \overset{M}{\underset{m=1}{\sum }}|a_{m}|$$

where *L* indicates the loss function to minimize. The first term represents the prediction risk, and the second part penalizes large values for the coefficients of the base learners.

Support Vector Regression (SVR) is an optimization problem of a convex loss function to be minimized to find, in such a way, the flattest zone around the function (known as the tube) that contains the most observations [14]. The convex optimization, which has a unique solution, is solved, using appropriate numerical optimization algorithms. The function to be minimized is the following:

$$\frac{1}{2}{\left\|\beta \right\|}_{2}^{2}+C\;\overset{N}{\underset{i=1}{\sum }}V_{\epsilon }\left(y_{i}-x_{i}\beta_{i}\right)$$

with

$$V_{\epsilon }\left(r\right)=\{\begin{array}{c} 0,\;\;\left|r\right|<\epsilon \\ \left|r\right|-\epsilon ,\;\;otherwise \end{array}$$

and *C* is an additional hyperparameter. The greater is *C*, the greater is our tolerance for points outside *ϵ*.

### 2.2. Classification Methods

Classification methods are applied when the response variable is binary or, more generally, categorical. Naive Bayes (NB) is a “probabilistic classifier” based on the application of the Bayes’ theorem with strong (naïve) independence assumptions between the features [15]. Indeed, NB classifier estimates the class *C* of an observation by maximizing the posterior probability:

$$\arg\underset{C}{\max}\frac{p\left(x|C\right)p\left(C\right)}{p\left(x\right)}$$

Support Vector Machine (SVM) builds a model that assigns new examples to one category or the other, making it a non-probabilistic binary linear classifier [16]. The underlying idea is to find the optimal separating hyperplane between two classes, by maximizing the margin between the closest points of these two classes. To find the optimal separating hyperplane it needs to minimize:

$$\underset{\beta }{\min}\frac{1}{2}\beta^{T}\beta \;subject\;to\;y_{i}\left(x_{i}^{T}\beta \right)\geq 1.\;for\;i=1,\ldots ,n$$

A quadratic programming solver is needed to optimize the aforementioned problem.

The k-nearest neighbor (KNN) is a non-parametric ML method which can be used to solve classification problems [17]. KNN assigns a new case into the category that is most similar to the available categories. Given a positive integer k, KNN looks at the k observations closest to a test observation $x_{0}$ and estimates the conditional probability that it belongs to class *j* using the formula

$$P\left(Y=j|X=x_{0}\right)=\frac{1}{k}\underset{i\;\in N_{0}}{\sum }I\left(y_{i}=j\right)$$

where $N_{0}$ is the set of *k* -nearest observations, and *I* is the indicator function, which is 1 if a given observation is a member of class j and 0 otherwise. Since the k nearest points are needed, the first step of the algorithm is calculating the distance between the input data points. Different distance metrics can be used; the Euclidean distance is the most used.

A Neural Network (NN) is a set of perceptrons (artificial neurons) linked together in a pattern of connections. The connection between two neurons is characterized by the connection weight, updated during the training, which measures the degree of influence of the first neuron on the second one [18]. NN can be also applied in unsupervised learning. Strengths and limitations of each approach are summarized in Table 1.

### 2.3. Supervised Machine Learning Approaches in Pharmacogenetics

Recent studies in pharmacogenetics aiming to predict drug response used a SML approach with satisfactory results (Table 2). In particular, a study assessing the pharmacogenetics of antidepressant response compared different supervised techniques such as NN, recursive partitioning, learning vector quantization, gradient boosted machine and random forests. Data involved 671 adult patients from three European studies on major depressive disorder. The best accuracy among the tested models was achieved by NN [19]. Another study on 186 patients with major depressive disorder aimed to predict response to antidepressants and compared the performance of RT and SVM. SVM reported the best performance in predicting the antidepressants response. Moreover, in a second step of the analysis, authors applied LASSO regression for feature selection allowing the selection of 19 most robust SNPs. In addition, application of SML allowed to distinguish remitters and non-remitters to antidepressants [20].

A field of pharmacogenetics where SML techniques find wide application is the study of the response to anti-cancer drugs. In this regard, EN, SVM and RF reported excellent accuracy, generalizability and transferability [21,22,23]. 

Studies on warfarin dosing applied different SML techniques (NN, RIDGE, RF, SVR and LASSO) showing a significant improvement in the prediction accuracy compared to standard methods [24,25,26,27]. Another study on warfarin stable dosage prediction using seven SML models (multiple linear regression, NN, RT, SVR and RF) showed that multiple linear regression may be still the best model in the study population [28].

A comparative study on prediction of various clinical dose values from DNA gene expression datasets using SML, such as RTs and SVR, reported that the best prediction performance in nine of 11 datasets was achieved by SVR [29]. Recently, an algorithm “AwareDX: Analysing Women At Risk for Experiencing Drug toxicity” based on RF was developed for predicting sex differences in drug response, demonstrating high precision [30].

## 3. Unsupervised Machine Learning Approaches

**Figure 3:**
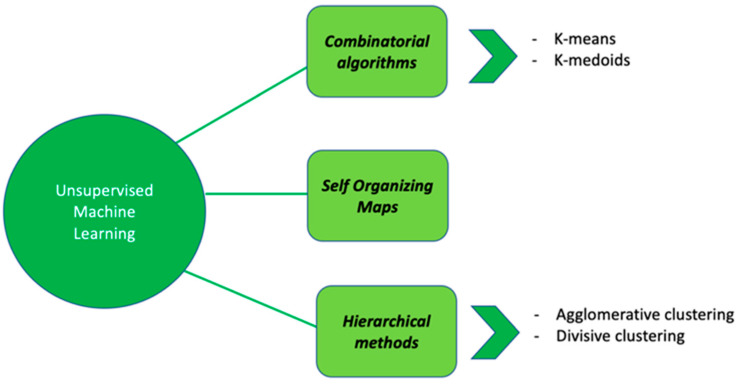
Summary representation of different UML algorithms: some examples.

Regarding UML, data-driven approaches by using clustering methods can be used to describe data with the aim of understanding whether observations can be stratified into different subgroups. Clustering methods can be divided into (i) combinatorial algorithms, (ii) hierarchical methods and (iii) self-organizing maps (Figure 3).

### 3.1. Combinatorial Algorithms

In combinatorial algorithms, objects are partitioned in clusters trying to minimize a loss function, e.g., the sum of the within clusters variability. In general, the aim is to maximize the variability among clusters and to minimize the variability within clusters. K-means is considered the most typical representative of this group of algorithms. Given a set of input variables $\left(x_{1},x_{2},\ldots ,\;x_{n}\right)$, k-means clustering aims to partition the n observations into k (≤n) sets S = $\left\{S_{1},S_{2},\ldots ,\;S_{k}\right)$, minimizing the within-cluster variances. Formally, the objective function to be minimized is the following:

$$L=\overset{k}{\underset{i=1}{\sum }}\underset{x_{j}\in S_{i}}{\sum }||x_{j}-\mu_{i\;}|{|}^{2}$$

where $\mu_{i}$ is the set of centroids in $S_{i}$. The k-means algorithm starts with a first group of randomly selected centroids, which are used as starting points for every cluster, and then performs iterative calculations to optimize the positions of the centroids. In k-means clustering, the centroids $\mu_{i}$ are the means of the cluster $S_{i}$. The algorithm stops if there is no change in the centroid or if a maximum number of iterations has been reached [31]. K-means is defined for quantitative variables and Euclidean distance metric; however, the algorithm can be generalized to any distance D. K-medoids clustering is a variant of K-means that is more robust to noises and outliers [32]. K-medoids minimizes the sum of dissimilarities between points labeled to be in a cluster and a point designated as the center of that cluster (medoids), instead of using the mean point as the center of a cluster.

### 3.2. Hierarchical Methods

Hierarchical clustering produces, as output, a hierarchical tree, where leaves represent objects to be clustered, and the root represents a super cluster containing all the objects [33]. Hierarchical trees can be built by consecutive fusions of entities (objects or already formed clusters) into bigger clusters, and this procedure configures an agglomerative method; alternatively, consecutive partitions of clusters into smaller and smaller clusters configure a divisive method.

Agglomerative hierarchical clustering produces a series of data partitions, $P_{n},\;P_{n-1},\ldots ,P_{1}$, where $P_{n}$ consists of n singleton clusters, and $P_{1}$ is a single group containing all n observations. Basically, the pseudo algorithm consists in the following steps:

1. Compute the distance matrix D;
2. The most similar observations are merged in a first cluster;
3. Update D;
4. Steps 2 and 3 are repeated until all observations belong to a single cluster.

One of the simplest agglomerative hierarchical clustering methods is the nearest neighbor technique (single linkage), in which the distance between clusters (r,s) is computed as follows:

$$D\left(r,s\right)=\underset{i\in r,j\in s}{\min}d\left(i,\;j\right)\;\;$$

At each step of hierarchical clustering, the clusters r and s, for which *D(r,s)* is minimum, are merged. Therefore, the method merges the two most similar clusters.

In the farthest neighbor (complete linkage), the distance between clusters (r,s)  is defined as follows:

$$\;D\left(r,s\right)=\underset{i\in r,j\in s}{\max}d\left(i,\;j\right)\;\;$$

At each step of hierarchical clustering, the clusters r and s, for which D(r,s) is minimum, are merged. 

In the average linkage clustering, the distance between two clusters is defined as the average of distances between all pairs of objects, where each pair is made up of one object from each group. 

Divisive clustering is more complex than agglomerative clustering; a flat clustering method as “subroutine” is needed to split each cluster until each data have its own singleton cluster [34]. Divisive clustering algorithms begin with the entire data set as a single cluster and recursively divide one of the existing clusters into two further clusters at each iteration. The pseudo algorithm consists in the following steps:

1. All data are in one cluster;
2. The cluster is split using a flat clustering method (K-means, K-medoids);
3. Choose the best cluster among all the clusters to split that cluster through the flat clustering algorithm;
4. Steps 2 and 3 are repeated until each data is in its own singleton cluster.

### 3.3. Self Organizing Maps

Self-Organizing Maps (SOM) is the most popular artificial neural network algorithm in the UML category [35]. SOM can be viewed as a constrained version of K-means clustering, in which the original high-dimensional objects are constrained to map onto a two-dimensional coordinate system. Let us consider n observations, M variables (dimensional space) and K neurons. Denoting by $w_{i}$, i=1…K, the position of the neurons in the M-dimensional space, the pseudo-algorithm consists in:

1. Choose random values for the initial weights $w_{i}$;
2. Randomly choose an object i and find the winner neuron j whose weight $w_{j}$ is the closest to observation $x_{i}$;
3. Update the position of $w_{j}$ moving it towards $x_{i}$;
4. Update the positions of the neuron weights $w_{h}$ with h $h\in \;{NN}_{j}\left(t\right)$ (winner neighborhood);
5. Assign each object i to a cluster based on the distance between observations and neurons.

In more detail, the winner neuron is detected according to:

$$w_{j}=\underset{i=1\ldots K}{\min}\|x-w_{i}\|$$

The winner weight updating rule is the following:

$$w_{j}\left(t+1\right)=w_{j}\left(t\right)+\eta \left(t\right)\|x-w_{i}\|$$

where η(t) is the learning rate which decreases as iterations increases, and the ${NN}_{j}\left(t\right)$ updating rule is the following:

$$w_{h}\left(t+1\right)=w_{h}\left(t\right)+f\left(NN_{j}\left(t\right),\;t\right)\|x-w_{h}\|$$

where the neighborhood function $f\left(NN_{j}\left(t\right),\;t\right)$ gives more weight to neurons closer to the winner i than to those further away. Strengths and limitations of each approach are reported in Table 3.

### 3.4. Unsupervised Machine Learning Approaches in Pharmacogenetics

Since the main goal in pharmacogenetics is to predict drug response, only few studies have used UML techniques (Table 4). These techniques have mainly been used for data pre-processing to identify groups. Indeed, Tao et al., to balance the dataset of patients treated with warfarin and improve the predictive accuracy, proposed to solve the data-imbalance problem using a clustering-based oversampling technique. The algorithm detects the minority group, based on the association between the clinical features/genotypes and the warfarin dosage. A new synthetic sample, generated selecting a minority sample and finding k-nearest neighbors of the minority sample, was added to the dataset. Then, two SML techniques (RT and RF) were compared in order to predict the warfarin dose. Both models (RT and RF) achieve the same or higher performance in many cases [36]. A study aiming to combine the effects of genetic polymorphisms and clinical parameters on treatment outcome in treatment-resistant depression used a two-step ML approach. First, patients were analyzed using a RF algorithm, while in a second step, data were grouped through cluster analysis. Cluster analysis allowed identifying 5 clusters of patients significantly associated with treatment response [37].

## 4. Conclusions

ML techniques are sophisticated methods that allow obtaining satisfactory results in term of prediction and classification. In pharmacogenetics, ML showed satisfactory performance in predicting drug response in several fields such as cancer, depression and anticoagulant therapy. RF proved to be the most frequently applied SML technique. Indeed, RF creates many trees on different subsets of the data and combines the output of all the trees, reducing variance and the overfitting problem. Moreover, RF works well with both categorical and continuous variables and is usually robust to outliers. 

Unsupervised learning still appears to not be frequently used. The potential benefits of these methods have yet to be explored; indeed, using UML as a preliminary step for the analysis of drug response could provide subgroups of response that are less arbitrary and more balanced than the standard definition of response.

Although ML methods have shown superior performances with respect to classical ones, some limitations should be considered. Firstly, ML methods are particularly effective for analyzing large complex datasets. The amount of data should be large to provide enough information for solid learning. Indeed, the small sample size may potentially affect the stability and reliability of ML models. Moreover, due to algorithm complexity, other potential limitations could be overfitting, the lack of standardized procedures and the difficulty of interpreting data.

The main strength of ML technique is to provide very accurate results, with a notable impact according to precision medicine principles.

In order to overcome the possible limitations of ML, future directions should be focused on the creation of an open-source system to allow researchers to collaborate in sharing their data. 

## Figures and Tables

**Table 1:** Supervised machine learning approaches: strengths and limitations.

Methods	Strengths	Limitations
GLM	The response variable can follow any distribution in the exponential family Easy to interpret	Affected by noisy data, missing values, multicollinearity and outliers
Ridge	Overcomes multicollinearity issues	Increased bias
LASSO	Avoids overfitting Effective in high dimensional settings	Selects only one feature from a group of correlated features
EN	Selects more than n predictors when n (sample size)<<p (# of variables)	Computationally expensive with respect to LASSO and Ridge
RT	Easy to implement Ability to work with incomplete information (missing values)	Computationally expensive
RF	High performance and accuracy	Less interpretability High prediction time
SVR	Easy to implement Robust to outliers	Unsuitable for large datasets Low performance in overlapping situations *
NB	Suitable for multi-class prediction problems	Independence assumption Assigns zero probability to category of a categorical variable in the test data set that was not available in the training dataset
SVM	Suitable for high dimensional settings	No probabilistic explanation for the classification Low performance in overlapping situations * Sensitive to outliers
KNN	Easy to implement	Affected by noisy data, missing values and outliers
NN	Robust to outliers Ability to work with incomplete information (missing values)	Computationally expensive

GLM: Generalized Linear Model; LASSO: Least Absolute Shrinkage and Selection Operator; EN: Elastic-net; RT: Regression Tree; RF: Random Forest; SVR: Support Vector Regression; NB: Naïve Bayes; SVM: Support Vector Machine; KNN: K-nearest Neighbor; NN: Neural Network; #: number; * overlapping can arise when samples from different classes share similar attribute values.

**Table 2:** Summary of the study using SML approaches.

Reference	AIM	Included Population	Methodologies	Results
Fabbri 2018 [19]	To predict response to antidepressants	671 patients	NN and RF	The best accuracy among the tested models was achieved by NN
Maciukiewicz 2018 [20]	To predict response to antidepressants	186 patients	RT and SVM	SVM reported the best performance in predicting the antidepressants response.
Kim 2019 [21]	To study of the response to anti-cancer drugs	1235 samples	EN, SVM and RF	Sophisticated machine learning algorithms allowed to develop and validate a highly accurate a multi-study–derived predictive model
Cramer 2019 [22]	To study of the response to anti-cancer drugs	1001 cancer cell lines and 265 drugs	linear regression models	The interaction-based approach contributes to a holistic view on the determining factors of drug response.
Su 2019 [23]	To study of the response to anti-cancer drugs	33,275 cancer cell lines and 24 drugs	Deep learning and RF	The proposed Deep-Resp-Forest has demonstrated the promising use of deep learning and deep forest approach on the drug response prediction tasks.
Ma 2018 [24]	To study the warfarin dosage prediction	5743 patients	NN, Ridge, RF, SVR and LASSO	Novel regression models combining the advantages of distinct machine learning algorithms and significantly improving the prediction accuracy compared to linear regression have been obtained.
Liu 2015 [25]	To study the warfarin dosage prediction	3838 patients	NN, RT, SVR, RF and LASSO	Machine learning-based algorithms tended to perform better in the low- and high- dose ranges than multiple linear regression.
Sharabiani 2015 [26]	To study the warfarin dosage prediction	4237 patients	SVM	A novel methodology for predicting the initial dose was proposed, which only relies on patients’ clinical and demographic data.
Truda 2021 [27]	To study the warfarin dosage prediction	5741 patients	Ridge, NN and SVR	SVR was the best performing traditional algorithm, whilst neural networks performed poorly.
Li 2015 [28]	To study the warfarin dosage prediction	1295 patients	Linear regression model, NN, RT, SVR and RF	Multiple linear regression was the best performing algorithm.

LASSO: Least Absolute Shrinkage and Selection Operator; EN: Elastic-net; RT: Regression Tree; RF: Random Forest; SVR: Support Vector Regression; SVM: Support Vector Machine; KNN: K-nearest Neighbor; NN: Neural Network.

**Table 3:** Unsupervised machine learning approaches: strengths and limitations.

Methods	Strengths	Limitations
K-means	Reallocation of entities is allowed No strict hierarchical structure	*A priori* choice of the number of clusters Dependent on the initial partition
K-medoids	Reallocation of entities is allowed No strict hierarchical structure	*A priori* choice of the number of clusters Dependent on the initial partition High computational burden
Agglomerative/ Divisive Hierarchical	Easy to implement Easy interpretation	Strict hierarchical structure Dependent on the updating rule
SOM	Reallocation of entities is allowed No strict hierarchical structure	*A priori* choice of the number of clusters Dependent on the number of iterations and initial weights

SOM: self-organizing maps.

**Table 4:** Summary of the study using UML approaches.

Reference	AIM	Included population	Methodologies	Results
Tao 2020 [36]	To balance the dataset of patients treated with warfarin and improve the predictive accuracy.	592 patients	Cluster analysis	The algorithm detects the minority group, based on the association between the clinical features/genotypes and the warfarin dosage.
Kautzky 2015 [37]	To combine the effects of genetic polymorphisms and clinical parameters on treatment outcome in treatment-resistant depression.	225 patients	Cluster analysis	Cluster analysis allowed identifying 5 clusters of patients significantly associated with treatment response.

## Data Availability

Not applicable.
